# Meniscus Matrix Remodeling in Response to Compressive Forces in Dogs

**DOI:** 10.3390/cells9020265

**Published:** 2020-01-21

**Authors:** Umberto Polito, Giuseppe M. Peretti, Mauro Di Giancamillo, Federica Boschetti, Liliana Carnevale, Maria C. Veronesi, Luca M. Sconfienza, Marco Agnoletto, Laura Mangiavini, Silvia C. Modina, Alessia Di Giancamillo

**Affiliations:** 1 Department of Veterinary Medicine, Università degli Studi di Milano, 26900 Lodi, Italy; umberto.polito@unimi.it (U.P.); mauro.digiancamillo@unimi.it (M.D.G.); lilianacarnevale@tiscali.it (L.C.); maria.veronesi@unimi.it (M.C.V.); alessia.digiancamillo@unimi.it (A.D.G.); 2 Department of Biomedical Sciences for Health, Università degli Studi di Milano, 20133 Milan, Italy; luca.sconfienza@unimi.it (L.M.S.); laura.mangiavini@unimi.it (L.M.); 3 IRCCS, Istituto Ortopedico Galeazzi, 20161 Milan, Italy; federica.boschetti@polimi.it (F.B.); marcoagno@hotmail.it (M.A.); 4 Department of Chemistry, Materials and Chemical Engineering “Giulio Natta”, Politecnico di Milano, 20133 Milan, Italy; 5 Department of Health, Animal Science and Food Safety, Università degli Studi di Milano, 26900 Lodi, Italy; silvia.modina@unimi.it

**Keywords:** meniscus, proteoglycans and glycosaminoglycans, cell–extracellular matrix interaction, extracellular matrix remodeling, GAGs, compression, Young’s compressive elastic modulus, dog

## Abstract

Joint motion and postnatal stress of weight bearing are the principal factors that determine the phenotypical and architectural changes that characterize the maturation process of the meniscus. In this study, the effect of compressive forces on the meniscus will be evaluated in a litter of 12 Dobermann Pinschers, of approximately 2 months of age, euthanized as affected by the quadriceps contracture muscle syndrome of a single limb focusing on extracellular matrix remodeling and cell–extracellular matrix interaction (i.e., meniscal cells maturation, collagen fibers typology and arrangement). The affected limbs were considered as models of continuous compression while the physiologic loaded limbs were considered as controls. The results of this study suggest that a compressive continuous force, applied to the native meniscal cells, triggers an early maturation of the cellular phenotype, at the expense of the proper organization of collagen fibers. Nevertheless, an application of a compressive force could be useful in the engineering process of meniscal tissue in order to induce a faster achievement of the mature cellular phenotype and, consequently, the earlier production of the fundamental extracellular matrix (ECM), in order to improve cellular viability and adhesion of the cells within a hypothetical synthetic scaffold.

## 1. Introduction

Menisci are C-shaped fibrocartilaginous wedge structures with a crucial and well-documented role in the knee joint, as reviewed by [1]. Load transmission across the joint [2,3], shock absorption [3,4,5], nutrient distribution [6,7,8], joint lubrication [8], improved congruency [9,10,11,12], proprioception [13,14,15,16,17,18,19,20], and nociception [17,18] are recognized as the main functions of the meniscus within the knee joint. These functions derive from the peculiar structure and composition of the tissue. Menisci may be divided into three morpho-functional different zones: the red–red zone, fully vascular and strictly in contact with the joint capsule; the white–white zone, completely avascular and corresponding to the inner sharp edge of the meniscus; and an intermediate zone, called the red–white zone, which presents midway characteristic of vascularization and cells phenotyping with the other two [11]. The different phenotypes of cells present throughout the tissue are distributed in a regional-specific manner and are responsible for the double nature of the meniscus. In the outer zone, fibrocyte-like cells reflect the fibrous pattern of the meniscus, while in the inner zone, chondrocyte-like cells are responsible for the cartilaginous-like behavior of the tissue. These regional variations are acquired during the latter stage of meniscal development, since initially cells show no phenotypical differentiation [21]. Menisci are mainly composed of water (72%); dry matter is organized in a collagen network, principally of type I and II, which entraps different phenotypes of cells in a rich-of-proteoglycans extracellular matrix (ECM) as reviewed by [21]. In pigs, collagen fibers are arranged in site-specific and depth-specific ways that allow the meniscus to withstand tensile forces [12], while proteoglycans (PGs), capturing and releasing water molecules via their negatively charged glycosaminoglycans (GAGs) terminuses, generate an osmotic pressure that allows menisci to withstand compressive forces as demonstrated by Mahmood et al. in bovine species [22]. Collagen fibers are mainly of type I and II, with a predominance of collagen type I all over the red–red zone and the co-expression of both collagens type I and II (40:60) in the white–white zone [21]. Type I fibers are principally disposed following the circumferential shape of the meniscus in the outer zone [23], while collagen type II fibers are mainly expressed, as the PGs, in the inner zone and in a radial direction, as observed in bovines and humans [23,24]. Collagen radial fibers envelop the circumferentially disposed collagen type I fibers to avoid their displacement when the application of a compressive force occurs [24]. The peculiar arrangement of collagen bundles and the strict interconnection between collagen fibers (particularly those of type II) and GAGs lead to the elastic and viscoelastic properties that characterize the meniscus as reviewed by [25]. Meniscal development is characterized by major remodeling processes as both cellularity and vascular supply continue to decrease, while GAGs increase in the inner zone and collagen fibers pass from a disorganized arrangement to the well-organized one as previously reviewed [26]. Furthermore, the collagen fibers present in the early stages of meniscal maturation are composed only of collagen type I; type II collagen will be expressed only in later stages of development and primarily in the inner zone, as demonstrated in sheep and pigs [27,28]. Postnatal weight-bearing and joint motion are the principal factors that determine the phenotypical and architectural changes occurring during the maturation process of the meniscus and are implied in determining the orientation of the collagen fibers in humans [1] and the production of GAGs in pigs [11]. Meniscal tears are reported as spontaneous or, more frequently, secondary to anterior cruciate ligament (ACL) deficiency in both human and canine species [29]. The dog is one of the most utilized animal models in meniscal pathology and repair due to the similarity in pathogenesis and anatomy between these two species [29,30]. Nowadays, different techniques are available to achieve meniscus repair (through sutures or implants) or the complete or partial replacement (with allograft or with scaffolds, either synthetic or biological) [31]. However, they do not avoid technical critical points. Tissue engineering targets to replace the damaged meniscus with a synthetic material that mimics its main morphology and functions [21]. This material should recapitulate the native tissue (with scaffolds and cells) or may just replicate its mechanical properties (only with scaffolds). Meniscus repair can be considered satisfactory when the chosen cells are able to overcome the mechanical forces that are commonplace in the knee and are able to retain their fibro-chondrogenic phenotype, to proliferate in and adhere to the site of injury [32,33,34,35,36]. On the other hand, the complete replacement of the meniscus is obtained by utilizing different supports, from silk to the recently developed polycarbonate-urethane (PCU)-polyethylene reinforced meniscal substitute. These materials are cell-free and present some advantages, such as the ease of use, the availability, and the relatively lower cost with respect to the cell-based regenerative medicine. Additionally, they can be considered a valuable option in the case when a prompt extended mechanical support represents the main issue, as in the case of great loss of material [36]. On the other hand, these biomaterials tend to be biodegraded [36]. The choice of living cell-laden biomaterials permits replenishing any extracellular matrix that has been degraded over time [36]. Given these premises, the development of biomaterials for meniscus tissue engineering has been investigated in parallel with tissue engineering efforts by using cells and biomaterials together and both these approaches cannot ignore the knowledge of the cells’ behavior under different types of stimulus. Thus, different stimuli, both biochemical (i.e., growth factors) and biomechanical, have been applied to enhance the production of meniscal ECM [21] focusing on the importance of this study for tissue engineering and, ultimately, for the clinic. In this context, this study aimed to understand the effect of compressive forces on the development and remodeling of neonatal meniscus structure, focusing on cell maturation and collagen fiber arrangement. The study has been performed on a litter of 12 Dobermann Pinschers, of approximately 2 months, euthanized since affected by the quadriceps muscle contracture syndrome of a single limb, a rare pathology characterized by the fixed hyperextension of both knee and ankle joints. Since these joints are characterized by a consensual mechanism of flexion/extension, the affected knee is unable to flex [37,38], making these joints a realistic model of compressive tissue engineering bioreactors.

## 2. Materials and Methods

### 2.1. Description of the Study

**Figure 1:**
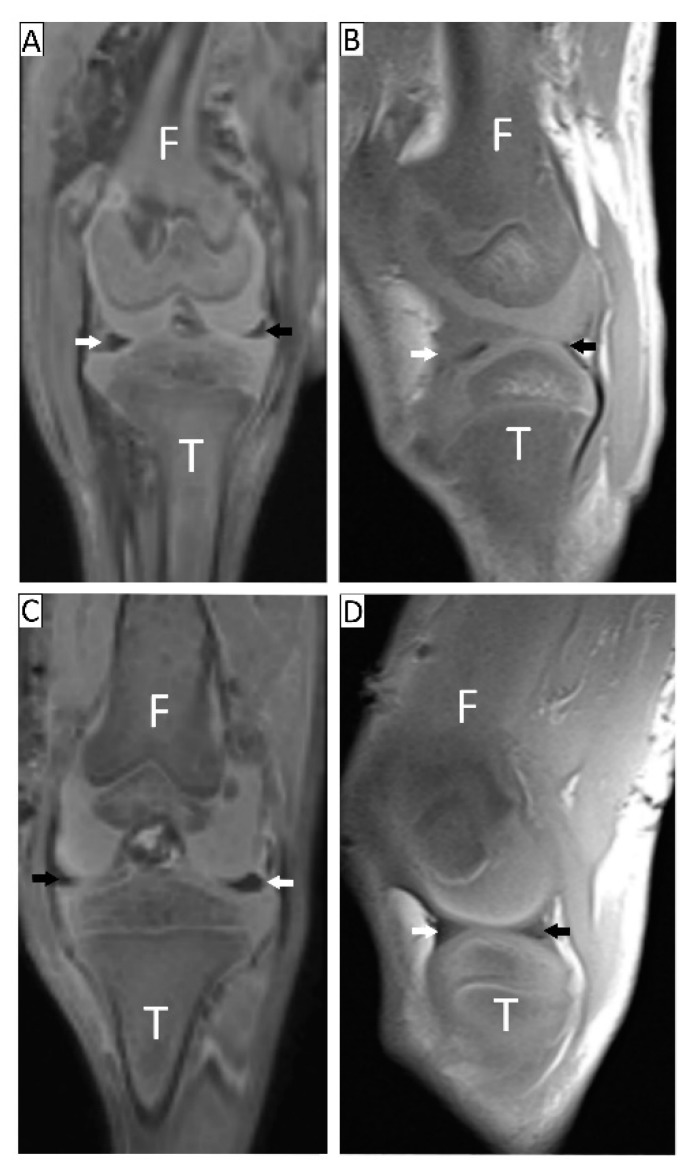
MR acquisition of a quadriceps-contracture-syndrome-affected knee and a healthy one. Coronal (**A**,**C**) and sagittal (**B**,**D**) reformat of 3D T1-weighted MR acquisition. **A**,**B**: right contracted limb; **C**,**D**: left noncontracted limb. The different morphology of menisci is clearly seen (white arrow, lateral meniscus body; black arrow, medial meniscus body; F, femur; T, tibia).

The menisci utilized in this study belonged to a litter of 12 Doberman Pinschers of 57 days old, euthanized for a severe quadriceps contracture after being admitted to the Veterinary Teaching Hospital of the Università degli Studi di Milano. No animals were sacrificed for the purposes of this study. All the animals used died for reasons that have no relationship with this study and the owner authorized, by means of an informed consent, the use of the carcasses for research purposes. The project was approved by the Ethic Committee of the Università degli Studi di Milano, Milan, Italy (OPBA, Protocol number 58/2016). The syndrome affected only the right hind limb in all the puppies and was manifested since the age of about 1 month, so that the left limb was used as control in all animals. The unit of the Obstetric and Gynecologic Clinic performed the puppies’ postmortem evaluation, sampling, and data registration. All the carcasses underwent an accurate postmortem evaluation and the hind limbs of the specimens were submitted to a radiological examination to exclude fractures and/or recent fracture healing processes and to evaluate knee joint appearance and alignment (Figure S1A–D). Two puppies out of 12, randomly chosen, underwent postmortem magnetic resonance (MR) for a better assessment of the soft tissues and of the menisci of both limbs (Figure 1). Finally, a lateral arthrotomy was performed and the joints were dissected to isolate lateral and medial menisci of both limbs. Thus, five animals were destined for morphological evaluations (histology, histochemistry, immunohistochemistry) and the other seven were destined for biomechanical and biochemical analyses. In the text below, we will refer to a “compressed” meniscus to describe menisci harvested from the right joints while menisci harvested from the left joints will be labeled as “healthy”.

### 2.2. Radiological and MR Analyses

Radiographic images of both hind limbs were acquired with a CR25 AGFA (S.p.A.) assembled with a radiological unit (Simply - Arcom S.r.l. - Italy) using a 0.6 mm focal spot. The focal spot–film distance was 100 cm. For each limb, ventro-dorsal and medio-lateral views were performed (Figure 1). MR images were acquired with a 1.5T system (Avanto, Siemens Healthineers, Forchheim, Germany) equipped with a phased-array, eight-channel coil. Routine T1-weighted, T2-weighted, and 3D T1-weighted sequences were performed. Three-dimensional sequences were then reformatted according to the preferred plan for best meniscal visibility.

### 2.3. Morphological Analyses: Macroscopic Evaluation, Histology, Histochemistry, and Double Immunohistochemistry

**Figure 2:**
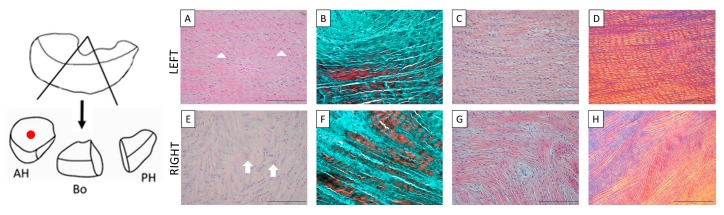
On the left, study design. The preparation of the meniscal samples in base of the type of technique performed is shown on the top. AH: anterior horn; BO: body; PH; posterior horn. Original picture by U.P. On the right, histological (**A** and **E**) and histochemical (**B**–**D** and **F**–**H**) staining of the left anterior horns of the left and right menisci (red dot); longitudinal section. **A**–**D**: Hematoxylin–eosin (**A**); Goldner–Massons’ trichrome (**B**); Picrosirius Red (**C**); and polarized light microscopy (**D**) of the left healthy meniscus; **E**–**H**: Hematoxylin–eosin (**E**); Goldner–Massons’ trichrome (**F**); Picrosirius Red (**G**); and polarized light microscopy (**H**) of the right compressed meniscus. Note the different arrangements of the collagen fibers between left and right menisci and the different shape of cells: fusiform (white arrowheads) in the left meniscus and rounded (white arrows) in the right one.

Both medial and lateral menisci were harvested from the right and left knees of five animals (total: 20 samples) and macroscopically examined. Successively, they were subdivided with two transversal cuts in three sections: the anterior and posterior horns and the central body (Figure 2; left side). Samples were immediately fixed in 10% buffered formalin, and then dehydrated in a graded series of ethanol, cleared with xylene, and embedded in paraffin. Serial longitudinal and transverse microtome sections (4 µm thick) were obtained from each sample. The differences in cellular maturation grade and matrix deposition between the two populations were evaluated through histology (hematoxylin–eosin staining) and histochemistry (Goldner–Masson’s trichrome staining, Sirius Red staining, and Safranin-O staining). Hematoxylin–eosin staining was performed to evaluate the potential morphological differences between compressed and uncompressed meniscus cells and the arrangement of the collagen fibers. Sirius Red staining and Goldner–Masson’s trichrome staining were chosen to highlight the collagen fiber deposition and arrangements. Safranin-O staining was performed to highlight the presence of GAGs within the tissue. Sirius Red histochemical-stained sections were analyzed by means of polarized light microscopy (Olympus BX51 light microscope Olympus, Opera Zerbo, Milan Italy, equipped with a digital camera) to assess the spatial orientation of the fibers, highlighted by the birefringence of collagen fibers. Double immunofluorescence was performed to reveal the localization and the possible colocalization of the two main expressed types of collagens (type I and II). After rehydration, a heat-induced antigen retrieval [11] and three times washing in PBS (pH 7.4) were performed and sections were successively incubated with the first-step primary antiserum, 1:50 collagen I (Abcam, Cambridge, UK) for 24 h at 18–20 °C, washed in PBS, and subsequently treated with the Avidin–Biotin blocking kit solution (Vector Laboratories Inc., Burlingame, CA, USA). The sections were then washed in PBS for 10 min and incubated with a solution of goat biotinylated anti-rabbit IgG (Vector Laboratories Inc., Peterborough, UK), 10 µg/mL in Tris-buffered saline (TBS) for 1 h at 18–20 °C. After rinsing twice in PBS, the sections were treated with Fluorescein–Avidin D (Vector Laboratories Inc., Peterborough, UK), 10 µg/mL in NaHCO3, 0.1 M, pH 8.5, 0.15 M NaCl for 1 h at 18–20 °C. For the second step of the double immunofluorescence procedure, sections were treated in a 2% hyaluronidase solution at room temperature for 30 min, subsequently incubated with 1:50 anti-collagen II antiserum (Chondrex Inc., Redmond, WA USA), rinsed in TBS for 10 min, and incubated with 10 µg /mL goat biotinylated anti-mouse IgG (Vector Laboratories Inc., Peterborough, UK) for 1 h at 18–20 °C. The sections were then washed twice in PBS, treated with Rhodamine–Avidin D (Vector Laboratories Inc., Peterborough, UK), 10 µg /mL in NaHCO3, 0.1 M, pH 8.5, with 0.15 M NaCl for 1 h at 18–20 °C, briefly washed in PBS, mounted in Vectashield Mounting Medium (Vector Laboratories Inc., Peterborough, UK), and observed with a Confocal Laser Scanning Microscope (FluoView FV300; Olympus, Hamburg, Germany) equipped with Argon/Helio–Neon–Green lasers with excitation and barrier filters set for fluorescein and rhodamine. Images containing superimposition of fluorescence were obtained by sequentially acquiring the image slice of each laser excitation or channel. In the double immunofluorescence experiment, the absence of cross-reactivity with the secondary antibody was verified by omitting the primary antibody during the first incubation step.

### 2.4. Biomechanics

The elastic modulus in compression (Ec) was evaluated by means of an EnduraTEC ELF^®^ 3200 machine (TA Instrument, New Castle, DE, USA), equipped with a 22N load cell. Medial and lateral menisci from both right and left knees of seven puppies were transversally sectioned in the anterior horn, body, and posterior horn as previously described (overall 84 samples), stored in saline solution NaCl 0.9%, and frozen at –80 °C until the time of testing. At least 24 h before test execution, samples were thawed at room temperature (23 °C). For each zone, a cylindrical part perpendicular to the femoral and tibial surfaces was cut using a die cutter. Before testing, dimensional measurements (diameter and height) of the specimens were made with a digital caliper. Samples were inserted into a Plexiglas cell containing PBS solution to avoid dehydration and their initial thickness was measured from the position of the testing machine actuator, after imposing a preload of approximately 0.01N. The sample was then subjected to a multi-ramps stress relaxation test, made of five increasing 4% strains at a velocity of 0.1%/s, followed by stress relaxation to equilibrium for 600 s. The compressive modulus, Ec, was obtained for each ramp from the equilibrium data as the ratio between values of relaxation stress and the corresponding values of strain.

### 2.5. Biochemical Analysis

Biochemical analysis was performed on the same samples previously analyzed by biomechanical tests. Meniscal portions were digested in a solution of 125 mg/mL of papain (Sigma-Aldrich, Milan, Italy) in 100 mM sodium phosphate, 10 mM sodium EDTA (Sigma-Aldrich, Milan, Italy), 10 mM cysteine hydrochloride (Sigma-Aldrich, Milan, Italy), 5 mM EDTA adjusted to pH 6.5 for 16–24 h at 60 °C. After the digestion, samples were stored at −80 °C until the analysis to assess proteoglycan and DNA contents. Proteoglycan content was estimated by quantifying the amount of sulphated glycosaminoglycans using the 1,9-dimethylmethylene blue dye binding assay (Polysciences, Inc., Warrington, PA, USA) and a microplate reader (wavelength: 540 nm). The standard curve for the analysis was generated by using bovine trachea chondroitin sulphate A (Sigma-Aldrich, Milan, Italy)). DNA content was evaluated with the Quant-iT Picogreen dsDNA Assay Kit (Molecular Probes, Invitrogen, Waltham, MA, USA) and a fluorescence microplate reader and standard fluorescein wavelengths (excitation 485 nm, emission 538 nm, cut-off 530 nm). The standard curve for the analysis was generated using the bacteriophage lambda DNA supplied with the kit.

### 2.6. Statistical Analysis

Statistical analyses of the biomechanical and biochemical data were analyzed with two-way ANOVA with side (left and right) and meniscal portions (anterior horn, body, posterior horn) as main factors using the general linear model of the SAS (version 8.1, Cary Inc., NC). The meniscus was considered to be the experimental unit of all response variables. The data were presented as least square means ± SEM. Differences between means were considered significant at *p* < 0.05 and highly significant at *p* < 0.01.

## 3. Results

### 3.1. Radiological and MRI Analyses

Postmortem examination confirmed the right unilateral quadriceps contracture in all dogs. The affected knee was unable to flex in the customary 90° angle. No evidence of fractures and/or fracture healing processes was revealed by X-ray examination of the right limb; however, a rotation of the proximal epiphysis and a torsion of the distal epiphysis of the femur and tibia, respectively, and a consequent malalignment of the knee (Figure S1A,B) were detected. No abnormalities were shown in the left hind limb (Figure S1C,D). MRI examinations revealed that menisci of the left limbs had physiological shape, while the right limbs’ menisci were less visible and different in shape (Figure 1).

### 3.2. Morphological Analyses: Macroscopic Evaluation, Histology, Histochemistry, and Double Immunohistochemistry

Macroscopic differences in menisci were observed: the left (healthy) menisci showed the expected semilunar and wedge-like shape that was not preserved in the right (compressed) menisci (Figure S1E). Differences were also detectable with histology, histochemistry, and double immunohistochemistry analyses: all the presented images were captured in the anterior horns in order to compare healthy and compressed menisci in the site of highest compression. Collagen fiber arrangement and cellular shape were evaluated by means of hematoxylin–eosin staining (Figure 2A,E), Goldner–Masson’s trichrome staining (Figure 2B,F), and Sirius Red staining (Figure 2C,G); the spatial orientation of the fibers, highlighted by the birefringence of collagen fibers, was observed by a polarized light microscopy after Sirius Red histochemical staining (Figure 2D,H). All over the healthy menisci, collagen fibers showed the typical wavy aspect, as they were composed of crimps. These crimps were well evident in the histologic (Figure 2A) and histochemical staining (Figure 2B,C). Collagen bundles followed a well-ordinated and unidirectional arrangement (Figure 2A–D); collagen fibers appeared well highlighted under the polarized light and showed the characteristic anisotropic behavior that allowed noting the fibers’ crimps (Figure 2D). On the contrary, the compressed meniscus was characterized by a chaotic distribution of the collagen fibers, which looked stretched without their typical wavy appearance (Figure 2E–H) and the anisotropic behavior (Figure 2H). Cells showed a fusiform shape in the healthy meniscus (Figure 2A; white arrowheads) and a more rounded shape in the compressed meniscus (Figure 2E; white arrows).

**Figure 3:**
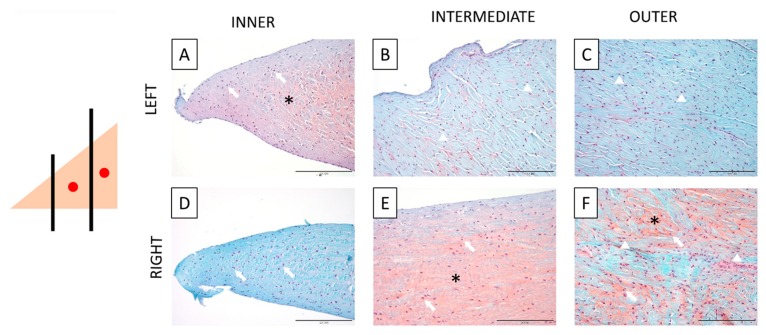
Histochemical Safranin-O staining. On the left, a schematic drawing that explains the type of section (transversal) and the three areas of the meniscus (inner, intermediate, and outer), with red dots showing the sites of compression, limited to the right meniscus. **A**–**C**: Staining of the inner (**A**), intermediate (**B**), and outer (**C**) zones of the left meniscus. **D**–**F**: Staining of the inner (**D**), intermediate (**E**), and outer (**F**) zones of the compressed meniscus. Note the differences between the localization of the GAGs (stained in orange, asterisk) in the matrix of the two menisci: in the inner zone for the healthy meniscus and in the intermediate and the outer zones for the compressed one, and the different shapes of cells’ nuclei: fusiform in the outer zones of the left meniscus and in the outermost area of the right one (arrowheads), and more rounded in the inner area of the left and right meniscus and in the more compressed intermediate and outer zone of the right meniscus (arrow). Left: healthy meniscus; right: compressed meniscus.

Safranin-O staining was performed to highlight the presence of GAGs within the tissue (orange/pink) and to evaluate the morphology of the cells in the three meniscal zones. The healthy meniscus showed a higher concentration of GAGs in the inner zone (Figure 3A, asterisks), while the outer zones presented only a sporadic staining for GAGs (Figure 3B,C); the cells of the inner zone showed a more rounded shape when compared with the cells of the other two zones, which were more fusiform (Figure 3A, arrows; vs. B and C, arrowheads). The compressed meniscus shows a higher concentration of GAGs in the two outer zones while the inner zone shows only a pale staining (Figure 3B,C vs A). Differently to what previously observed in the healthy knee meniscus, cells have a rounded shape in all the three areas (Figure 3D–F; arrows), with a higher incidence of fusiform cells only in the outermost zone (Figure 3F; arrowheads).

**Figure 4:**
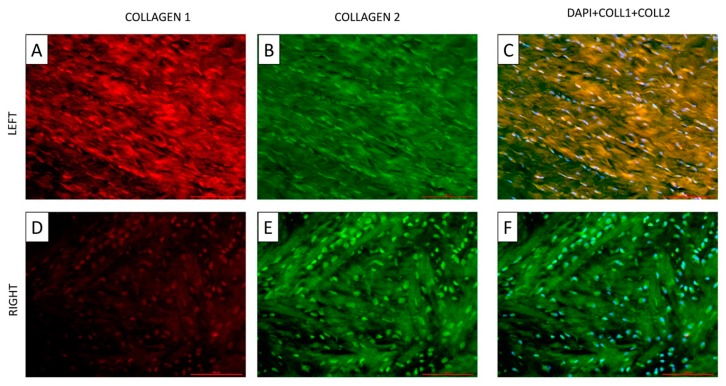
Double immunohistochemistry of the anterior horns of the left and right menisci (see Figure 2 for compression site); longitudinal section. **A** and **D**: Collagen type I expression in left (**A**) and right (**D**) menisci. **B** and **E**: Collagen type II expression in left (**B**) and right (**E**) menisci. **C** and **F**: Co-expression of collagen type I and II in left (**C**) and right (**F**) menisci. Note the round shape of the nuclei and the random arrangement of the collagen fibers present in the right menisci (**D–F**) vs. the elongated nuclei and the well-organized arrangement present in the left ones (**A**–**C**). Red: collagen type I; green: collagen type II; yellow: co-expression of collagen types I and II; light blue: DAPI. Left: healthy meniscus; right: compressed meniscus.

Immunohistochemistry was performed to evaluate the expression of the two principal types of collagens (I and II) present in the menisci as well as the cellular morphology (Figure 4A–F). Collagen type I (Figure 4A, marked in red) and II (Figure 4B, marked in green) were co-expressed in the matrix and presented a reduced nuclear expression in the physiological meniscus (Figure 4C). Nuclear shape (Figure 4A–C) was elongated and recalls the aspect previously described with histologic and histochemical staining. The compressed meniscus was characterized by a predominance of collagen II (Figure 4F) and a clear nuclear localization (Figure 4E,F). Collagen I was slightly expressed both in the matrix and nuclei (Figure 4D). The nuclei of the compressed menisci had a more rounded shape (Figure 4D–F) with respect to the elongated nuclei present in the healthy ones (Figure 4A–C).

### 3.3. Biomechanics

**Figure 5:**
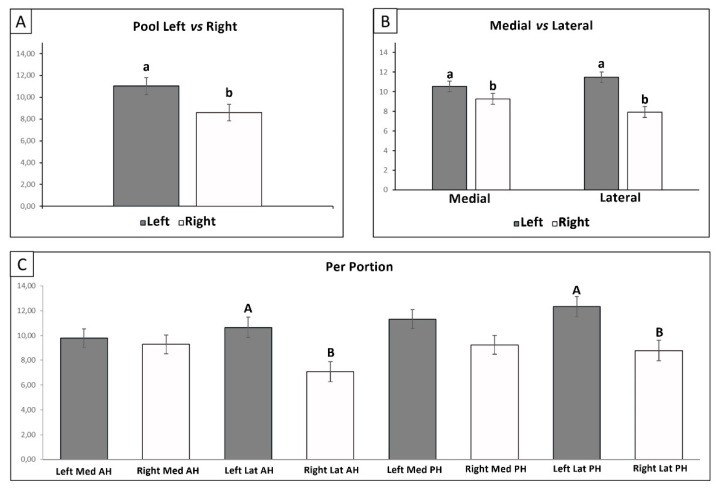
Biomechanical evaluation of the Young’s Elastic Modulus in compression (EC). **A**: comparison between pooled left and right menisci’s EC; **B**: Comparison between the EC of pooled medial and lateral menisci; **C**: EC comparison among all the different meniscal portions. Med: medial meniscus; Lat: lateral meniscus; AH: anterior horn; PH: posterior horn; gray: left/healthy menisci; white: right/compressed menisci. a, b: *p <* 0.05; A, B: *p* < 0.01.

Unconfined compressive elastic modulus (Ec) in response to compression was tested in order to assess eventual differences between lateral and medial menisci (Figure 5B) and between the anterior and posterior horns (Figure 5C), always considering the difference between compressed and healthy menisci (Figure 5A). Pooled left (healthy) menisci showed a higher Ec with respect to the right (compressed) ones (*p* < 0.05). Medial and lateral healthy menisci showed a higher Ec confronted with the counterparts of the compressed ones (*p* < 0.05 for both the comparisons). However, only the difference between the horns of the lateral menisci were statistically significant with the healthy menisci portions that showed the higher moduli values (for both, anterior and posterior horns, *p* < 0.01), while, in the medial compartment, no statistically significant results were shown considering the two horns. Nevertheless, in all the comparisons, the healthy meniscus showed the highest Ec.

### 3.4. Biochemical Analysis

**Figure 6:**
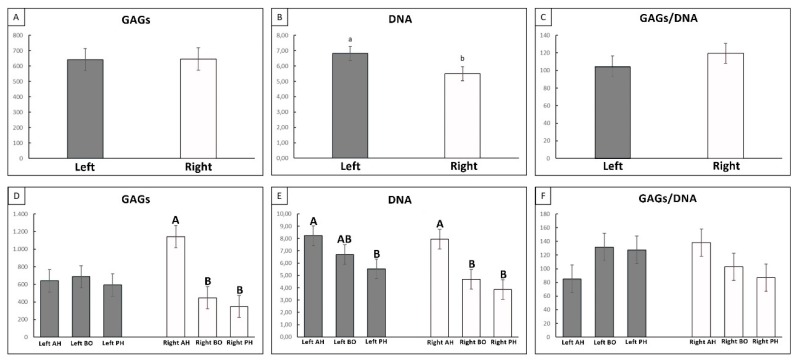
Biochemical assays: GAGs (**A**,**D**), DNA (**B**,**E**), and GAGs/DNA ratio (**C**,**F**) analysis for the whole left and right menisci (**A**,**B**,**C**) and for the different portions (**D**,**E**,**F**). Gray: left/healthy meniscus. White: right/affected meniscus. a, b: *p* < 0.05; A, B: *p* < 0.01.

The presence of GAGs (Figure 6A) and the quantification of cells (DNA; Figure 6B) within the meniscus were analyzed by means of biochemical assays, always considering the two sides. The GAGs/DNA ratio (Figure 6C) was also calculated to highlight the grade of maturation of the cells in relation to their capacity to produce GAGs in the ECM. GAGs quantification revealed a higher concentration of these proteins in the left (healthy) menisci with respect to the right one (compressed), though with no statistically significant differences (Figure 6A). A decrease in cell number was also seen, with a significant statistical difference (*p* < 0.05) in the right (compressed) menisci (Figure 6B). The GAGs/DNA ratio showed no statistical difference, but there was a higher value in the menisci harvested from the right knee (Figure 6C). When the different portions of each side’s meniscus are considered, two trends are principally shown: in the left (healthy) meniscus, no difference among anterior horn, body, and posterior horn was found (Figure 6D; left side), while in the compressed meniscus, a dichotomic pattern was present, in which the anterior horn showed a higher GAGs concentration with respect to the other two portions (Figure 6D; right side; *p* < 0.01). This dichotomic pattern was maintained even in the cellularity results of the compressed meniscus (Figure 6E; right side), as opposed to the results of the healthy menisci characterized by a smother differentiation among the three areas, with a higher abundance in the anterior horn with respect to the posterior horn and an intermediate value expressed in the body section (Figure 6E; left side). Intriguingly, the GAGs/DNA ratio showed a higher value in the anterior horn of the compressed meniscus (with respect to the other two portions) while its left counterpart showed the smaller value (Figure 6F); however, the differences described represent only trends, since none of these showed statistical significance.

## 4. Discussion and Conclusions

In this study, we evaluate the postmortem effects of constant and chronic mechanical stimuli on the development and cell–extracellular matrix interaction and remodeling of menisci in young dogs affected by a disease that induces the alteration of the functional mechanics of the knee. The pathology, which usually affects growing animals, causes the deformation and shortening of the affected limb. The deformation is a consequence of the increase of the bone segments’ length associated to the articular block and leads to an extra-rotation of the femoral distal epiphysis. In this way, a constant compression, that derives both from the longitudinal growth of the bone segments and from the weight bearing, is applied upon the structures which reside within the knee. On the other hand, tensile forces generated by the flexion/extension of the joint are practically zeroed. Due to the particular conformation of the deformed femur, the most compressed areas in the affected limbs end up being the external area of the menisci, contrarily to what is observed in the normal ones, in which the most compressed area is the inner zone. This condition was considered as a vital bioreactor that applies a dynamic compressive force (the alternation of load- and no-load-bearing) in association with a static compression (due to the continuous growth of the bone segments [39], that compressed the meniscus between the femur condyles and the tibial plateau), thus, menisci in this study were always subjected to a kind of compressive force, with no effective relax periods. Mechanical stresses are fundamental for the health and function of the meniscus [29] in both humans and animal models [40] and biomechanical stimuli are essential factors in the maturation of meniscal cells phenotype and in ECM meniscal remodeling, as demonstrated in pigs and sheep [28,41]. Lack of movement leads to disuse atrophy and loss of proteoglycan in mature menisci as reviewed by [42], and to the degeneration and the disappearing of the menisci during embryologic development, as observed in chickens [43]. Meniscal cells may react to mechanical stimulation either with an anti-inflammatory or proinflammatory response. Moreover, biomechanical stimulation influences the balance of ECM turnover toward anabolism or catabolism: these opposite behaviors depend on magnitude, frequency, duration, and type (static or dynamic) of the applied force [42,43]. Dynamic compression at 10% strain may stimulate both anabolic and catabolic metabolism with the suppression of proinflammatory mediators as reviewed by [42,44]. On the contrary, clear pathologic effects that lead to proteoglycans breakdown have been reported after the application of higher strain (20%) with upregulation of matrix metalloproteinase (MMP)-1, MMP-3, MMP-13, ADAMTS-4, inducible nitric oxide synthase (NOS2), interleukin-1α (IL-1α) mRNA, and nitric oxide (NO) as reviewed by [42]. Pathological models of abnormal joint loading resulted in catabolic and proinflammatory responses with a significant decrease in cell viability and a trend toward increased proinflammatory mediators, such as NO, as seen in the leporine model [45]. Nevertheless, studies on overloading models reported no macroscopic damage to the tissue, no detrimental effect on the sulphated-glycosaminoglycan (sGAG) content and release, the downregulation of many catabolic and proinflammatory genes, and no effect on the mechanical properties of the tissue. However, overloading caused large regions of cell death at the surface of the tissue [46]. Finally, Ballyns and Bonassar (2011) reported that a dynamic unconfined compression load (15% strain and 1 Hz three times/week for up to six weeks) led to an enhancement of the extracellular matrix content and compressive modulus after two weeks [47] and a more mature matrix (characterized by increase in collagen bundle formation, GAGs content, collagen content, and compressive modulus) was obtained by Puetzer and colleagues (2012) after two weeks of loading followed by four weeks of static culture [48]. In the present work, we cannot quantify the entity of the compression and the frequency of the dynamic compression due to the alternate periods of weight bearing and non-loading. However, the added value of the work is that we utilized tissue explants, which provide a reservoir of natural committed cells that preserved their natural relationship with and within the extracellular matrix. For these reasons, the effect of the compressive force was estimated without any bias that may be related to a synthetic composition or to the lack of the ECM. The morphological evaluation performed by Safranin-O staining confirmed that GAGs are produced in the most compressed areas: physiologically, the inner area of the meniscus is considered as the most compressed, and richer in GAGs [27]; however, due to the extra-rotation and the deformation of the distal femoral epiphysis consequent to the quadriceps contracture, a lateral shifting of the point of higher compression to the meniscal outer zones can be suggested and seems to be confirmed by the highest concentration of GAGs. Moreover, our morphological evaluation showed that cells react to the compressive stimulus by triggering their maturation: in the over-compressed area of the meniscus, cell nuclei acquired a rounded shape that is typical of meniscal fibro-chondrocytes, the phenotype that physiologically resides in the inner and cartilaginous-like portion of the meniscus; furthermore, these cells produce a quantity of GAGs larger than that produced by the cells of the uncompressed areas of the same samples and by the physiologically loaded cells of the counterparts. The apparently contrasting round-shaped cells present in the inner zone (and the fusiform-shaped cells present in the outermost area of the compressed meniscus) may be explained by an initial physiological regionalization of the tissue as previously described [1,21] that may have been interrupted since the pathology occurred. Additionally, an ischemic condition due to the stable compression may be hypothesized; this may have led the cells toward the cell phenotype that better survives in the hypoxic conditions: the chondrocyte-like one. Concerning the expression and the arrangement of collagen fibers, our histological and histochemical results showed how, in the healthy menisci, the collagen fibers displayed an ordered arrangement which follows the hypothetical tensile force generated by normal locomotion. Conversely, in the compressed menisci, the compressive force triggered a remodeling of the collagen fibers, which, in this case, showed a chaotic and not functional arrangement. Moreover, the typical wavy aspect of collagen fibers, characterized by the presence of crimps, responsible for the elastic properties of the meniscus, was displayed only in the physiologically loaded menisci, while the over-compressed ones were composed of stretched fibers that had lost their crimps and their elastic ability. We observed differences by the analyses of the collagen fibers, since we found that those derived from the healthy menisci were almost entirely composed of collagen type I and II as it is described in physiologic menisci of young sheep [27] and pigs [28], while the compressed ones were almost entirely composed of collagen type II. All these data were supported and quantified by the biomechanical and biochemical assays. Biomechanical evaluation of the meniscal elastic modulus in compression demonstrated the inability of the compressed menisci to withstand the compressive force, with respect to the healthy ones: Ec moduli of the compressed menisci were lower than those of the healthy ones, when side (left vs. right), compartments (medial or lateral), and portions (anterior or posterior horns) were considered. Biochemical quantification of meniscal tissue depicted a complex scene in which no differences were seen in the quantity of GAGs produced in the whole tissue with respect to the sides, but a significant decrease in cell density (DNA) and a higher GAGs/DNA ratio, although not statistically different, were observed. This may be considered as an initial switch toward a mature tissue (characterized by a small amount of cells that produce a rich ECM [11]). This pattern was mainly expressed in the anterior horn (with a dichotomic differentiation between anterior horn and the other two portions) that probably corresponded, in the compressed menisci, to the most compressed area. Taken together, the results of the biomechanical and biochemical analyses suggest that even if the over-compressed meniscus shows a precocious maturation of the resident cells (fibro-chondrocytic-like appearance) and consequently of the matrix (i.e., collagen type II expression, higher GAGs quantity), the lack of a well-organized collagen network leads to the development of an incompetent tissue. The importance of the collagen network in response to the application of tensile forces through the generation of the so-called hoop stress has been previously described [29] as well as the deep association of collagen type II fibers and GAGs [49]; however, to our knowledge, this is the first study that demonstrated the role of the collagen/GAGs relationship in the achievement of the typical anisotropic and elastic behavior of meniscal tissue in response to a compressive stimulus. Moreover, this investigation shows how native meniscal tissue varies under different biomechanical stimuli, producing useful background information for further tissue engineering applications. The results of this study suggest that a continuous compressive force, applied to the native meniscal cells, triggers a remodeling of the extracellular matrix and an early maturation of the cellular phenotype, at the expense of the proper organization of collagen fibers. The application of a compressive force for a limited time could be useful in meniscal tissue bioengineering in order to trigger a faster achievement of the mature fibro-chondrocyte phenotype from committed meniscal cells, and, consequently, the earlier production of the fundamental ECM, in order to improve cellular viability and adhesion of these cells within a potential synthetic scaffold. In this regard, this investigation shows how native meniscal tissue varies under different biomechanical stimuli, producing valuable background information for further tissue engineering applications. The results of this study suggest that a continuous compressive force, applied to the native meniscal cells, triggers a process of remodeling of the extracellular matrix and an early maturation of the cellular phenotype, at the expense of the proper organization of collagen fibers. For these reasons, we can envision the potential of a bioreactor that could generate compressive and tensile forces and so contribute to the maintenance of the phenotypical traits, but it can also replicate the meniscal maturation as it occurs in nature and, therefore, favors the development of meniscal substitutes before implanting cell-based scaffolds in vivo.

## Supplementary Materials

The following are available online at https://www.mdpi.com/2073-4409/9/2/265/s1, Figure S1: Radiological examination of the healthy and compressed hind limbs and macroscopic evaluation of the respective menisci.
