# Supplementary figures and images for: Meniscus Matrix Remodeling in Response to Compressive Forces in Dogs
Source: Cells. 2020 Jan 21;9(2):265. doi: 10.3390/cells9020265 (PMC7072134; doi:10.3390/cells9020265)

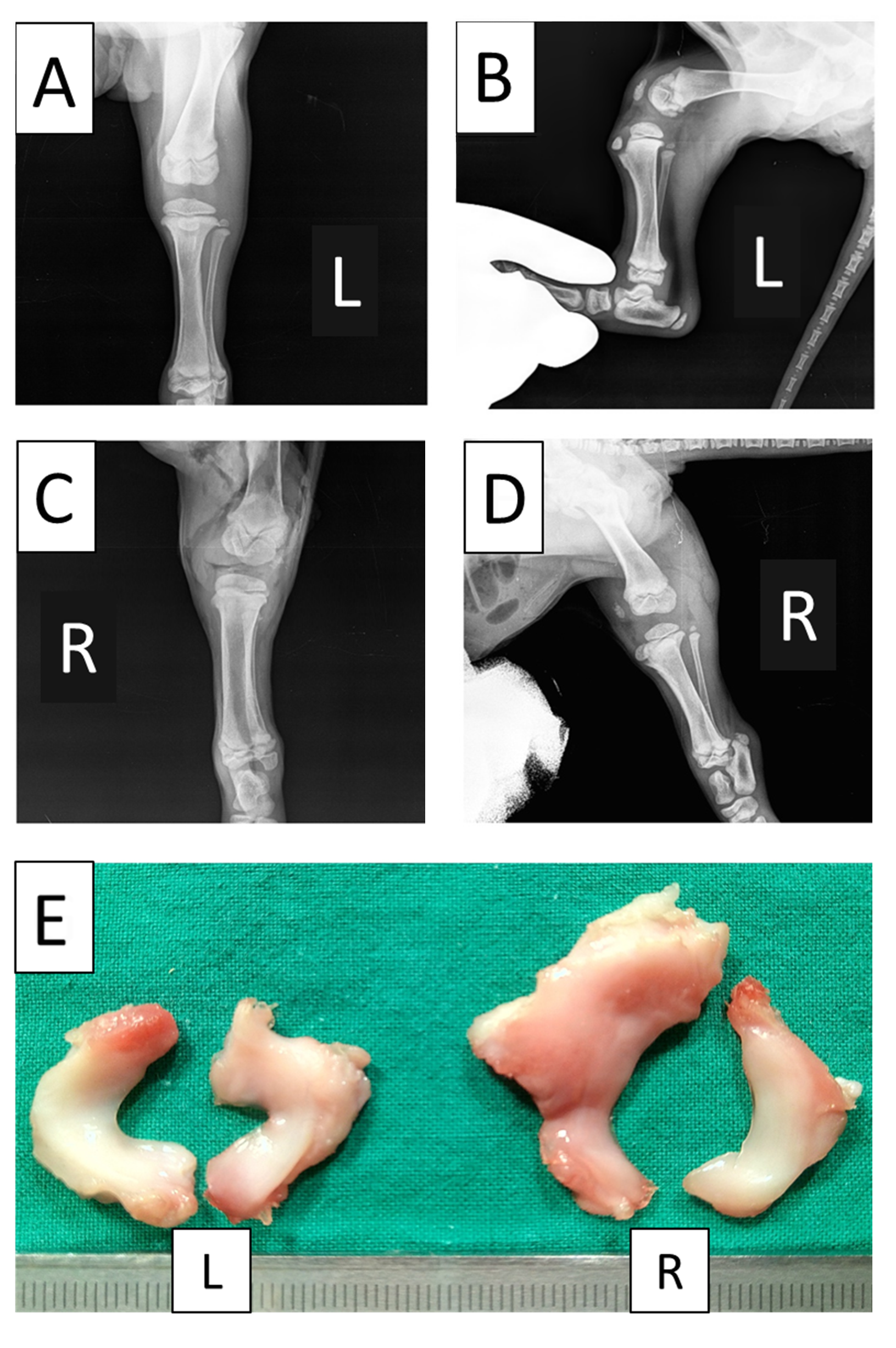

Supplement: Supplementary file 1 [file cells-09-00265-s001.tif]
